# Genetic Dissection and Functional Validation of *DXS2* Controlling Antheraxanthin Content in Maize Kernels

**DOI:** 10.3390/ijms27156770

**Published:** 2026-07-28

**Authors:** Xiuyi Fu, Hui Fang, Chen Chen, Chuanyong Chen, Shanshan Wu, Huasheng Zhang, Chunyuan Zhang, Xiaochen Li, Yanke Peng, Liya Guan, Siwen Bian, Feifei Chen, Qiang Cao, Baohua Wang, Yuandong Wang

**Affiliations:** 1 Maize Research Institute, Beijing Academy of Agriculture and Forestry Sciences (BAAFS), Beijing 100097, China; fuxiuyi@baafs.net.cn (X.F.); chenc71@163.com (C.C.); chuanyongchen@126.com (C.C.); wushanshan2017@126.com (S.W.); kobezhang24@163.com (H.Z.); zhangchunyuan@baafs.net.cn (C.Z.); 15801377950@163.com (X.L.); lgqianmo@163.com (Y.P.); 15711431637@163.com (L.G.); biansw1003@163.com (S.B.); maizefeifei@126.com (F.C.); caoqiang@nwafu.edu.cn (Q.C.); 2 College of Life Sciences, Nantong University, Nantong 226007, Chinabhwang@ntu.edu.cn (B.W.)

**Keywords:** maize, antheraxanthin, QTL mapping, *DXS2*

## Abstract

Antheraxanthin (ANT) is an important xanthophyll carotenoid in maize, but its genetic basis in maize kernels remains poorly understood. Here, we investigated the genetic architecture of ANT content using a teosinte–maize BC_2_F_5_ population evaluated across multiple environments. ANT showed abundant phenotypic variation and high broad-sense heritability (H^2^ = 0.86), indicating strong genetic control. A total of 11 QTLs were identified on chromosomes 1, 4, 5, 7, and 8, among which the major-effect locus *L4* on chromosome 7 was consistently detected across all environments and explained up to 37.46% of phenotypic variation. Fine mapping and candidate gene analysis identified *DXS2*, encoding 1-deoxy-D-xylulose 5-phosphate synthase 2, which is a key rate-limiting enzyme in the MEP pathway, as the strongest causal gene underlying *L4*. Functional validation using a MuDR insertion mutant demonstrated that loss of *DXS2* function significantly reduced ANT accumulation in maize kernels. Transcriptomic analysis further revealed that *DXS2* influences the MEP pathway, carotenoid biosynthesis, and downstream metabolic networks. These findings elucidate the genetic architecture of ANT accumulation, support *DXS2* as a key regulator connecting the MEP pathway with downstream carotenoid and ANT metabolism, and provide valuable genetic resources and theoretical support for carotenoid biofortification in maize breeding.

## 1. Introduction

Maize (*Zea mays* L.) is one of the most important staple crops across the world, providing food, protein, and feed for billions of people [1,2,3,4]. In addition to food, maize kernels accumulate various bioactive compounds that benefit human health, among which carotenoids and their derivatives have received lots of research attention [5,6]. Carotenoids are important phytonutrients with strong antioxidant activity, and are linked to low risks of chronic diseases, such as some cancers, cardiovascular diseases, and age-related macular degeneration [7,8,9]. However, carotenoid concentrations in modern maize varieties are often low and highly variable, highlighting the need to dissect the genetic basis of carotenoid accumulation to support effective biofortification.

Antheraxanthin (ANT; C40H56O3; chemically known as 5, 6-epoxy-5, 6-dihydro-β, β-carotene-3, 3′-diol) is a wide variety of plant-derived metabolite with strong antioxidant, anti-inflammatory, and anti-carcinogenic properties [10]. Carotenoids in maize kernels mainly consist of carotenes (e.g., β-carotene) and xanthophylls (e.g., lutein, zeaxanthin, and antheraxanthin), which differ substantially in their biological functions and nutritional significance [9]. β-Carotene, a major provitamin A carotenoid, has received considerable attention in biofortification efforts due to its contribution to vitamin A nutrition [11,12]. Lutein and zeaxanthin are the predominant xanthophylls in maize and are associated with antioxidant activity and human eye health [13,14]. In contrast, ANT is a less extensively characterized xanthophyll generated through the epoxidation of zeaxanthin within the xanthophyll cycle. Its genetic regulation and natural variation in crop species remain largely unexplored.

Recent studies have demonstrated that manipulation of carotenoid biosynthetic pathways can enhance the nutritional quality of maize kernels. For instance, marker-assisted breeding enabled the development of sweet corn lines co-enriched for β-carotene and anthocyanins, with β-carotene up to 10.23 μg/g and cyanidin-3-glucoside up to 8.10 mg/g [15]. Exogenous application of 24-epibrassinolide is also found to lead to higher carotenoid accumulation and antioxidant capacity in germinated maize kernels [16]. The biosynthetic genes and natural variation in β-carotene, lutein, and zeaxanthin have been extensively characterized [11,12,17]. In contrast, the genetic architecture controlling ANT accumulation remains largely unknown, and the loci and causal genes responsible for its natural variation in maize kernels have not been systematically dissected.

The methylerythritol 4-phosphate (MEP) pathway is the plastid-localized route for isoprenoid precursor synthesis, which supports the production of carotenoids, tocopherols, and other plastid isoprenoids [18,19,20]. 1-deoxy-D-xylulose 5-phosphate synthase (DXS) catalyzes the first committed and rate-limiting step of the MEP pathway, and its activity strongly controls metabolic flux into downstream isoprenoid biosynthesis [21,22]. Plants typically contain multiple *DXS* paralogs that have undergone functional diversification. In *Arabidopsis* and other plant species, *DXS1* is primarily associated with primary metabolism and supports general isoprenoid production for growth and development, whereas *DXS2* is preferentially involved in specialized isoprenoid biosynthesis, including carotenoid and terpenoid accumulation [23,24]. *DXS3* is a more divergent paralog, and emerging evidence indicates that it may have tissue-specific expression and distinct regulatory functions rather than simply acting as a redundant copy of *DXS1* or *DXS2* [25]. These functional differences among DXS paralogs suggest that individual DXS genes may differentially influence metabolic flux depending on developmental context and metabolic demand. In maize, *DXS2* has emerged as a promising candidate underlying natural variation in kernel carotenoid accumulation. A transposable element insertion in teosinte was shown to enhance *DXS2* expression and was subsequently selected during maize domestication and improvement [5]. However, whether *DXS2* specifically regulates ANT accumulation, and how it influences genome-wide metabolic pathways remain largely unknown.

Linkage mapping based on biparental populations has long served as the primary strategy for dissecting nutritional quality traits in maize. This approach has been widely employed to characterize the genetic basis of kernel carotenoids, oil, protein, starch, and related nutritional components [5,26,27,28,29,30]. Most kernel nutritional traits are quantitatively inherited and governed by numerous small-effect loci, and their phenotypic values are often heavily modulated by environmental variation, presenting substantial challenges for genetic dissection. Conventional QTL mapping frequently suffers from instability across environments, small individual effect sizes, and limited mapping resolution, which hinder the efficient identification of causal genes and the development of functional markers for molecular breeding [28,31,32]. To address these constraints, meta-QTL analysis has been applied in maize to detect stable QTLs with improved resolution for polygenic nutritional traits. This strategy has been shown to refine target genomic regions by up to 4.86-fold and to identify candidate genes functioning in grain filling, metal homeostasis, and fatty acid biosynthesis [33]. Moreover, the application of high-density molecular markers and multi-environment phenotypic evaluations further improve the reliability and accuracy of QTL detection. Together, these advances reinforce the value of integrating high-resolution linkage mapping, multi-environment trials, and functional validation to systematically resolve the complex genetic architecture of nutritional quality traits in maize.

In this study, we performed genetic and functional analyses of kernel ANT content using a BC_2_F_5_ population (TM population) derived from a cross between the maize inbred line Mo17 and the teosinte accession *Zea mays* ssp. *mexicana*. Our goals were to: (i) evaluate phenotypic variation and heritability of ANT across multiple environments; (ii) detect QTLs and epistatic interactions controlling ANT accumulation; (iii) fine-map a major-effect locus on chromosome 7 and identify the underlying gene; and (iv) functionally validate the role of *DXS2* in regulating ANT using a MuDR insertion mutant and transcriptome profiling. Our findings clarify the genetic control of ANT accumulation in maize kernels and suggest *DXS2* as a key regulator connecting the MEP pathway to downstream carotenoid and ANT metabolism, providing valuable information for the biofortification of nutritionally improved maize varieties.

## 2. Results

### 2.1. Phenotypic Variation in ANT in TM Population

**Figure 1:**
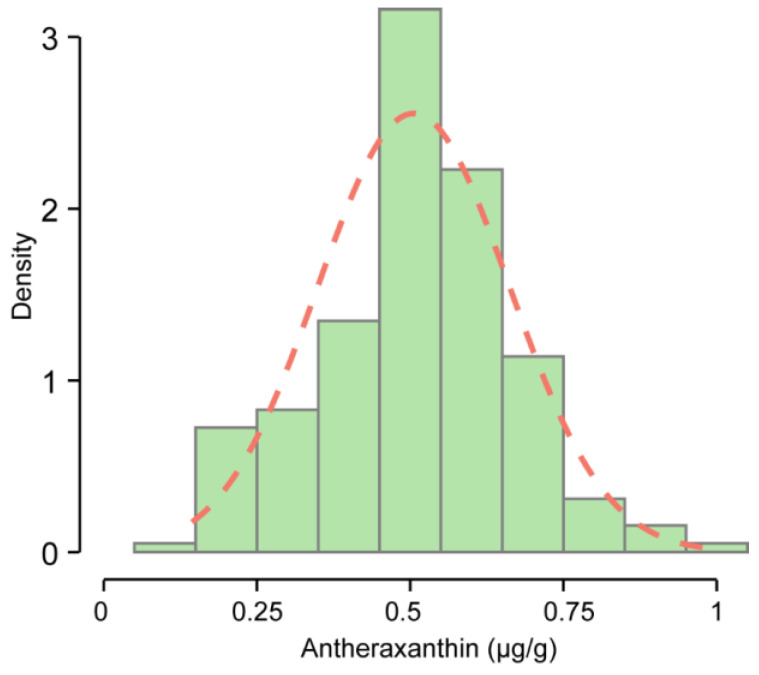
Distribution of antheraxanthin (ANT) content in maize kernels from the TM population. Light green bars show the density histogram of measured values and the red dashed line represents the fitted density curve.

The TM population exhibited abundant phenotypic diversity for kernel ANT content, with a continuous and approximately normal distribution (Figure 1). Based on the best linear unbiased prediction (BLUP) values across environments, ANT content in the TM population averaged 0.51 ug/g and ranged from 0.14 to 0.99 ug/g, representing an approximately 7-fold difference. Across the three individual environments, mean ANT values ranged from 0.40 to 0.63 ug/g, with difference fold ranging from 12.07 to 27.77 (Table 1). Pairwise phenotypic correlations across environments were high, varying from 0.66 to 0.81. Broad-sense heritability (H^2^) was 0.86, indicating that genetic effects predominantly determined ANT content in this population.

### 2.2. Linkage Mapping Dissect the Genetic Architecture of ANT Trait

**Figure 2:**
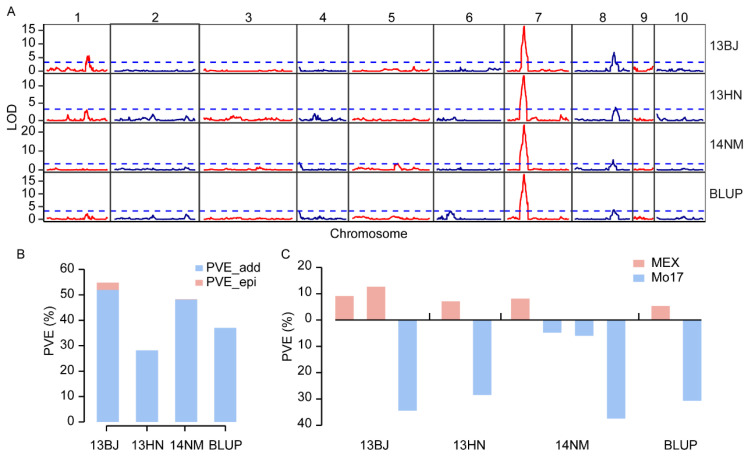
QTLs identified for ANT content using a high-density linkage map in the MT RIL population. (**A**) The distribution of ANT QTLs identified across the entire genome in different environments. (**B**) The total PVE by single (sky-blue bars) and epistatic (pink bars) QTLs for ANT trait. (**C**) Effect size (represented by PVE) and allelic origin of the increasing alleles for the identified single QTLs. Pink and sky-blue bars indicate that increasing alleles were derived from MEX and Mo17, respectively.

A genome-wide significance threshold (LOD = 3.3) for ANT content was determined by 1,000 permutation tests at *p* < 0.05. Using a high-density bin map, a total of 11 QTLs for the ANT were identified across three environments and BLUP values in the TM population, with two to four QTLs detected in each environment. These QTLs were distributed across five genomic regions on chromosomes 1, 4, 5, 7 and 8, with confidence intervals spanning from 1.43 to 43.59 Mb (Figure 2A, Table 2). The phenotypic variation explained (PVE) by individual QTLs ranged from 4.81% (*qANT5*) to 37.46% (*qANT7-3*), with a mean of 16.75%. The total PVE for all QTLs detected in each environment ranged from 28.16% to 51.94% (Figure 2B, Table S2). Of the five genomic regions, only locus *L4* on chromosome 7 represented a major effect and stable locus detected across all environments. Locus *L5* on chromosome 8 was also stable across environments, whereas the other three loci were environment-specific, each detected in only one environment. Notably, teosinte alleles at 45.45% of the QTLs (5/11) conferred increased ANT content (Figure 2C), indicating that teosinte harbors favorable alleles for this trait.

Beyond individual QTLs, two epistatic QTL pairs were detected: one between *qANT1* and *qANT7-1* in Beijing (2013), and another between *qANT5* and *qANT8-3* in Neimeng (2014). These epistatic interactions explained 0.1% and 2.9% of the phenotypic variation, respectively. Collectively, additive and epistatic effects accounted for 28.16% to 54.84% of the total phenotypic variation (Figure 2B; Table S2).

### 2.3. DXS2 Was Identified as a Candidate Gene Regulating ANT Content

**Figure 3:**
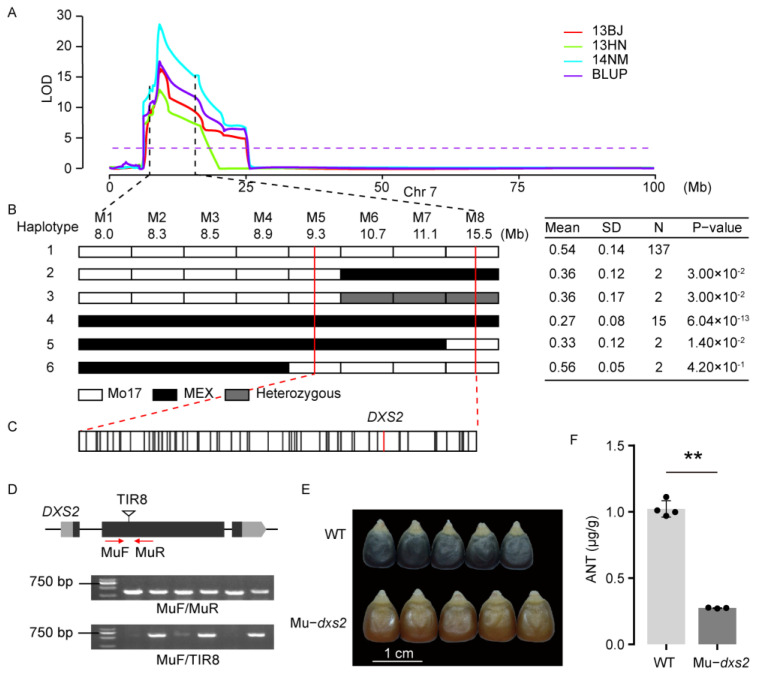
*DXS2* regulates ANT content in maize kernels. (**A**) LOD profile of the identified QTLs for *L4*. (**B**) Haplotype analysis of the *L4* interval with the ANT content BLUP value. Haplotypes with limited sample sizes (*N* ≤ 2) were interpreted cautiously because their phenotypic estimates may be affected by sampling variation. (**C**) Genes expressed in the endosperm within the refined *L4* interval. The red line indicates the location of the candidate gene, *DXS2*. (**D**) The gene structure of *DXS2* and insertion sites of MuDR transposons. Introns are represented by solid lines, exons by dark gray boxes, and untranslated regions (UTRs) by light gray boxes. Inverted triangles indicate transposon insertion sites. (**E**) Kernel phenotype of wild*−*type and Mu*−dxs2* mutants. (**F**) ANT content in kernels from wild*−*type and Mu*−dxs2* plants. “**” represents *p* < 0.01.

A major-effect locus, *L4* on chromosome 7, was consistently detected for ANT across all environments, explaining 28.49–37.46% of the phenotypic variation (Figure 3A; Table 2). High-density SNPs at this locus enabled the determination of recombination breakpoints between the two parents. The initial interval of *L4* spanned 7.5 Mb between markers M1 and M8, with all associated QTLs showing LOD scores greater than 10 (Figure 3A). Eight polymorphic SNPs defined six haplotypes in this region, four of which contained clear recombination breakpoints. Based on BLUP values, ANT content was significantly lower in haplotypes 2–5 (0.27–0.36 μg/g) than in haplotype 1 (0.54 μg/g), which carried Mo17 alleles across the entire region (Figure 3B). In contrast, haplotype 6 did not differ significantly from haplotype 1. These results delimited *L4* to a 6.2 Mb region containing 65 kernel-expressed genes, most of which were transcribed in endosperm (Figure 3C; Figure S1). Within this region, *DXS2* encodes 1-deoxy-Dxylulose-5-phosphate synthase 2, which catalyzes glyceraldehyde 3-phosphate and pyruvate to produce 1-deoxy-D-xylulose-5-phosphate in the methylerythritol phosphate (MEP) pathway [23]. Noticeably, *DXS2* is highly expressed during late endosperm development (Figure S1) and was previously shown to regulate total carotenoid content [5], highlighting it as the strongest candidate gene for *L4*. To further verify the role of *DXS2* in regulating ANT content, a MuDR transposon insertion mutant (Mu*−dxs2*) was obtained, with the insertion located in the second exon of *DXS2*. Homozygous mutants were confirmed using two pairs of gene-specific primers (Figure 3D, Table S1). Mutant kernels showed paler color and significantly lower ANT content than the wild type (Figure 3E,F). Beyond ANT content, six other carotenoid-related traits were also significantly altered in the mutant (Figure S2). These results demonstrate that the *L4* encodes DXS2.

### 2.4. Identification of Molecular Perturbation Mediated by DXS2

**Figure 4:**
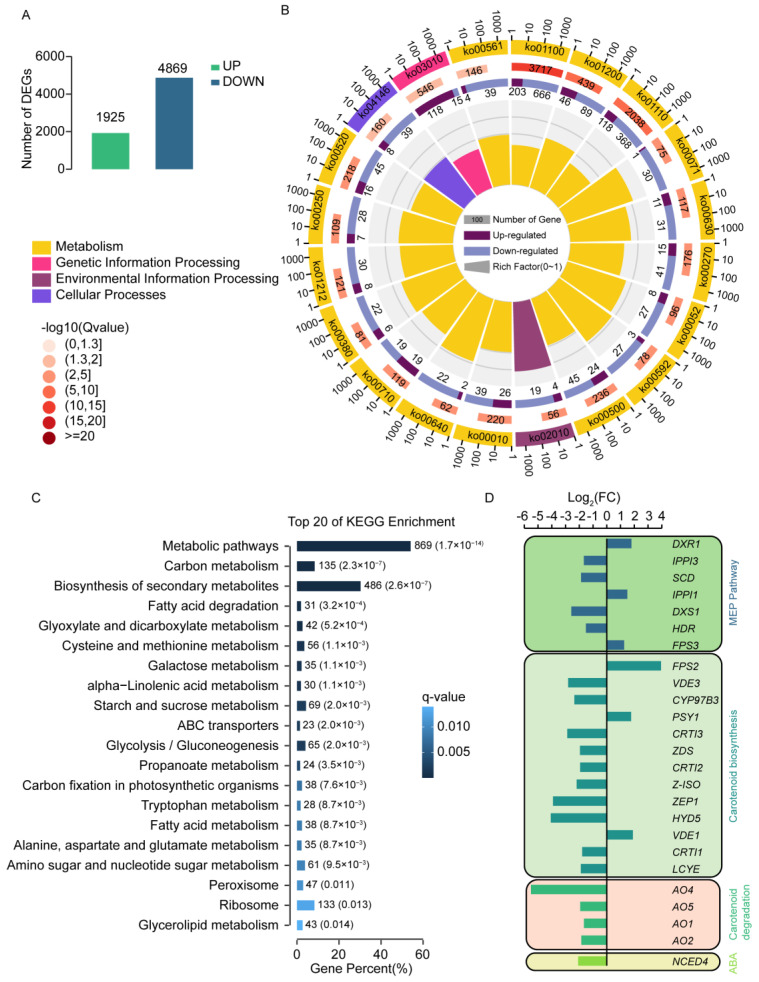
Transcriptomic overview of 20-DAP endosperms following loss of DXS2 function. (**A**) Number of DEGs between wild*−*type and Mu*−dxs2* mutants identified by RNA-seq. (**B**) KEGG pathway enrichment circle plot. The first circle: The top 20 enriched pathways, with the scale of the number of differentially expressed genes outside the circle. Different colors represent different A classes; The second circle: The number of this pathway in the background of differentially expressed genes and the Q value. The longer the bar, the more the number of differentially expressed genes in the background, and the smaller the Q value, the redder the color; The third circle: The bar chart of the proportion of upregulated and downregulated differentially expressed genes. Dark purple represents the proportion of upregulated differentially expressed genes, and light purple represents the proportion of downregulated differentially expressed genes; The specific values are shown below; The fourth circle: The RichFactor value of each pathway (the number of differentially expressed genes in this pathway divided by the total number of this pathway), background grid lines, each grid represents 0.1. (**C**) Bar chart of the top 20 enriched KEGG pathways. The *y*-axis shows pathway names, and the *x*-axis shows the rich factor (number of DEGs in the pathway/total genes in the pathway). Bar color intensity corresponds to the statistical significance (Q-value); darker colors indicate greater significance. The number of DEGs and the Q-value are labeled for each pathway. (**D**) Expression changes in genes involved in MEP and downstream pathways.

To further characterize transcriptomic changes mediated by *DXS2* dysfunction, RNA-seq was performed using 20-DAP endosperms from wild*−*type and Mu*−dxs2* plants, generating a total of 36 Gb of raw data (6 Gb per sample). Global transcriptome analysis identified 6,794 differentially expressed genes (DEGs; |log_2_FC| ≥ 1, FDR < 0.05), including 1,925 upregulated and 4,869 downregulated genes (Figure 4A; Figure S3). Loss of *DXS2* function resulted in extensive transcriptional changes, with a predominance of downregulated genes. As a key enzyme in the MEP pathway, DXS2 dysfunction broadly perturbed multiple metabolic pathways. These downregulated genes may include not only enzymes from various metabolic pathways but also their transcriptional regulators. KEGG enrichment revealed that 17 of the top 20 significantly enriched terms were associated with metabolic processes, including carbon metabolism, secondary metabolite biosynthesis, amino acid metabolism, starch and sucrose metabolism, and fatty acid metabolism (Figure 4B,C). These results indicate that disruption of *DXS2* alters broad transcriptional responses involving the MEP pathway and interconnected metabolic processes. The three remaining enriched terms were related to transport and translation, suggesting additional cellular responses associated with DXS2 dysfunction (Figure 4C).

We further examined genes involved in the MEP pathway and related pathways, including carotenoid synthesis and degradation, and ABA biosynthesis. A total of 26 genes showed significant differential expression in these pathways, including seven genes in the MEP pathway, 13 genes associated with carotenoid synthesis, four genes involved in carotenoid degradation and one gene related to ABA synthesis (Figure 4D). Among these genes, 73.08% (19/26) were sharply downregulated in Mu*−dxs2* mutants. Collectively, loss of *DXS2* function may repress the MEP pathway and downstream metabolic branches, thereby reducing accumulation of ANT and other carotenoid derivatives (Figure S2). These transcriptional changes may represent downstream responses associated with altered metabolic flux caused by *DXS2* dysfunction.

## 3. Discussion

### 3.1. Genetic Dissection of Antheraxanthin Accumulation in Maize Kernels

Maize kernel antheraxanthin (ANT) is a highly valuable carotenoid derivative with strong antioxidant, anti-inflammatory, and anti-carcinogenic properties [10], but its genetic structure and regulatory mechanisms in maize kernel remain largely unclear. In this study, we conducted a high-resolution linkage map using the TM BC_2_F_5_ population and detected a major and stable QTL for ANT on chromosome 7. We narrowed the locus to a 6.3 Mb interval, and functionally validated *DXS2* as the causal gene for ANT accumulation. Transcriptomic profiling further revealed that *DXS2* acts as a key regulator coordinating the MEP pathway, carotenoid metabolism, and a wide range of downstream metabolic networks. These findings clarify the genetic architecture of ANT in maize kernels and offer new gene targets and theoretical support for carotenoid biofortification in maize breeding.

Kernel carotenoid-related traits are typically quantitative traits and strongly influenced by environmental variation [5,6]. In this study, ANT content in the TM population exhibited high broad-sense heritability (H^2^ = 0.86) and strong phenotypic correlations across environments, indicating that genetic factors predominantly determine phenotypic variation. We detected 11 QTLs for ANT across multiple environments and BLUP values. Most QTLs were minor and environment-specific, whereas only the locus on chromosome 7 was consistently detected across all test environments, representing a major-effect QTL. Comparison with previously reported carotenoid QTLs showed that most ANT QTLs overlap with known regions associated with carotenoid content [6,27,28], This observation suggests that the genetic regulation of ANT accumulation is partially shared with the overall carotenoid biosynthetic pathway, likely reflecting the coordinated metabolic flux among different carotenoid components. However, *qANT1* has not been reported in previous studies, suggesting a potentially ANT-specific regulatory locus. Fine mapping and functional validation of this region may reveal the genetic mechanisms underlying ANT content in maize kernels. Two epistatic QTL pairs were identified, but contributed little to phenotypic variation, suggesting that additive effects constitute the primary genetic basis of ANT, while epistatic interactions play a minor role. These results are consistent with the genetic architecture of total carotenoids, β-carotene, and other nutritional quality traits in maize kernels [2,5,6,28,34]. The limited epistasis observed may result from the genetic structure of the BC_2_F_5_ population and the metabolic nature of this trait. The highly homozygous background after repeated backcrossing and selfing may reduce detectable locus–locus interactions. Additionally, ANT accumulation is directly linked to carotenoid biosynthesis, and variation in key pathway genes may generate stronger additive effects by altering metabolic flux. Similar genetic architectures have been reported for other maize nutritional traits, where major-effect loci contribute predominantly to phenotypic variation [6,35,36]. Notably, approximately 45.45% of the positive additive alleles for ANT were contributed by teosinte, supporting the view that teosinte retains numerous favorable alleles for nutritional quality that were lost or weakly selected during maize domestication and improvement [5].

### 3.2. DXS2 Regulates ANT Accumulation Through the MEP Pathway

The MEP pathway provides essential isoprenoid precursors for carotenoid biosynthesis, and DXS catalyzes the first rate-limiting step of this pathway. In this study, *DXS2* was located within the major QTL interval on chromosome 7 and showed high expression during late endosperm development. Loss of *DXS2* function through a MuDR insertion mutant significantly reduced ANT content, supporting its important role in ANT accumulation. This result is consistent with previous evidence that *DXS2* contributes to total carotenoid variation in maize kernels [5], and extends its potential function to the regulation of a specific epoxy carotenoid component (ANT). Transcriptomic analysis revealed that *DXS2* disruption resulted in extensive changes in gene expression, including genes involved in the MEP pathway, carotenoid biosynthesis and degradation, while slightly affecting ABA biosynthetic genes. These transcriptional responses are likely secondary consequences of altered metabolic states caused by impaired *DXS2* function, rather than evidence of direct transcriptional regulation by *DXS2*. KEGG enrichment also indicated that *DXS2* dysfunction affects multiple metabolic processes, including carbon metabolism, secondary metabolism, and energy-related pathways, reflecting the central role of the MEP pathway in plastidial isoprenoid metabolism [37]. Thus, *DXS2* may influence ANT accumulation primarily by controlling precursor availability and triggering downstream metabolic responses, rather than by directly coordinating multiple pathways.

### 3.3. Distinct Detection Efficiency Between Linkage Mapping and GWAS

The causal variant underlying *DXS2* function for ANT accumulation is a transposable element (TE) insertion located in the first intron. Our previous work demonstrated that this TE insertion underwent strong selection during maize domestication and improvement, and reached a high frequency or near fixation in yellow maize inbred lines (96.8%, 422/436) [5]. Consistent with this observation, analysis of the GWAS panel showed that the favorable *DXS2* TE insertion allele is present at a high frequency, resulting in limited allelic variation at this locus. The reduced polymorphism likely explains why GWAS failed to detect a significant association signal for ANT content in this region (Figures S4 and S5), although linkage mapping successfully identified *DXS2* as the strongest causal gene underlying this QTL. Because GWAS relies on segregating sequence variants to establish marker-trait associations, loci with strongly selected and highly frequent alleles may have reduced statistical power [38,39,40,41,42,43]. In contrast, the biparental TM population used for linkage mapping recovered the ancestral polymorphism at *DXS2*, and enabled effective detection of the major QTL. Thus, our results highlight that loci subjected to strong selection during domestication and breeding may become difficult to identify through GWAS in modern germplasm panels, but can be effectively resolved using linkage populations derived from diverse genetic backgrounds such as maize × teosinte. This finding underscores the value of wild relatives for revealing selected alleles that have become rare or fixed in cultivated maize germplasms.

### 3.4. Breeding Implications and Prospects for Maize Nutritional Improvement

As an antioxidant carotenoid with potential benefits for human health, increasing ANT content may contribute to improving the nutritional quality of maize [7,8,10]. The identification of *DXS2* and its favorable allele provides a potential genetic target for marker-assisted selection and carotenoid biofortification. Introgression or targeted modification of favorable *DXS2* alleles may contribute to enhancing ANT and carotenoid accumulation in maize [13]. The *DXS2*-mediated regulatory network revealed by transcriptomic analysis provides insights into the metabolic regulation of carotenoid biosynthesis and may offer a basis for future metabolic engineering strategies. However, because DXS2 catalyzes the first committed and rate-limiting step of the plastidial MEP pathway, altering its activity could influence flux toward multiple plastid-derived isoprenoid compounds, not only carotenoids. Therefore, potential pleiotropic effects and metabolic trade-offs should be carefully evaluated before its application in breeding. Future studies combining favorable *DXS2* alleles with other carotenoid-related genes may provide a potential strategy for balanced nutritional improvement in maize.

Nevertheless, several limitations should be acknowledged in this study. First, although genetic and mutant-based evidences support the contribution of *DXS2* to ANT accumulation, functional validation was based on a single MuDR insertion mutant. Additional validation using CRISPR/Cas9-mediated knockout, complementation, or overexpression approaches would further strengthen the functional evidence for *DXS2*. Second, although the major QTL interval was substantially narrowed, the fine-mapped interval still spans approximately 6.3 Mb and contains additional annotated genes. Thus, complementation experiments and functional characterization of natural *DXS2* alleles will be valuable for further evaluating the contribution of *DXS2* to ANT variation. Third, although the current results support *DXS2* as a major contributor to ANT accumulation, direct biochemical characterization of enzyme activity differences among natural *DXS2* alleles was not performed. Future studies using heterologous expression or purified protein studies will help determine whether natural allelic variation directly affects DXS2 enzymatic activity. Finally, this study was conducted using a single teosinte-derived backcross population. Validation across diverse maize genetic backgrounds will be necessary to further assess the broader contribution of *DXS2* to natural variation in ANT accumulation.

## 4. Materials and Methods

### 4.1. Plant Materials and Field Trials

The teosinte–maize (TM) population used in this study was derived from a cross between the elite maize inbred line Mo17 and the teosinte accession *Zea mays* ssp. *Mexicana* to introduce abundant genetic variation from teosinte while maintaining an improved maize genetic background, followed by two generations of backcrossing and repeated self-pollination to produce the BC_2_F_5_ population [5,44,45]. This population provides a suitable resource for identifying genetic factors controlling ANT accumulation with reduced background variation. The 191 TM lines were planted in a randomized complete block design with one replicate per environment. Field experiments were conducted at three environments, including Beijing (2013), Hainan (2013), and Inner Mongolia (2014). Each plot consisted of a single 2.5 m row with 0.67 m between rows, at a planting density of 45,000 plants ha^−1^. All plants were self-pollinated to ensure sufficient kernel yield, and ears were harvested at physiological maturity. From each line, well-developed ears with uniform maturity were selected, and kernels were dried to a constant weight in a ventilated oven at 45 °C before metabolite extraction [5,6].

### 4.2. Quantification of Antheraxanthin in Maize Kernels

Antheraxanthin was extracted and quantified using a modified high-performance liquid chromatography (HPLC) method [5,46,47]. Briefly, 50 fully dried kernels were ground into a fine powder using a high-throughput tissue grinder. Carotenoids were extracted using acetone containing 0.1% butylated hydroxytoluene (BHT) to inhibit oxidation. Following centrifugation, the supernatant was filtered through a 0.22 μm organic filter and analyzed using a Prominence HPLC unit (Agilent Technologies, California, USA). Chromatographic separation was performed on a YMC^®^ C30 reversed-phase column (250 × 4.6 mm, 5 μm) maintained at 30 °C. The mobile phase comprised methanol-water (95:5, *v*/*v*) and methyl tert-butyl ether, with a flow rate of 1.0 mL min^−1^. Quantification was achieved by standard regression with external standards (Sigma-Aldrich, Burlington, USA). All samples were analyzed in two technical replicates, and mean values were used for subsequent genetic analysis. Samples showing inconsistent technical replicates were re-extracted and re-measured.

### 4.3. Statistical Analysis of Phenotypic Data

Analysis of variance (ANOVA) was performed using the R software (v4.4.1) to evaluate the effects of genotype, environment, and their interaction on ANT content. Broad-sense heritability (H^2^) was calculated as: H^2^ = σg^2^/(σg^2^ + σge^2^/e + σε^2^/re), where σg^2^ = genetic variance, σge^2^ = genotype × environment interaction variance, σε^2^ = residual error variance, e = number of environments, and r = number of replicates [48]. Best linear unbiased prediction (BLUP) values across environments were calculated using linear mixed model with genotype and environment as random effects, implemented in the R package lme4 [6,28].

### 4.4. Genotyping and Construction of High-Density Bin Map

Genomic DNA was extracted from young leaf tissues using a modified CTAB protocol [49]. A total of 191 RILs and their parents were previously genotyped with the Illumina MaizeSNP50 BeadChip array [50]. From the total marker set, 12,390 high-quality SNPs were retained and partitioned into 1,282 recombinant bins to construct a high-density bin map. The final genetic map spanned 1,748 cM, with an average bin interval of 1.36 cM between adjacent bins [5,44]. This bin map was used for subsequent QTL mapping and epistatic interaction analyses.

### 4.5. Quantitative Trait Locus (QTL) Mapping

QTL analysis was performed using composite interval mapping (CIM) in Windows QTL Cartographer v2.5 [51]. Model 6 of the Zmapqtl module was implemented with a 2.0 cM scanning step and a 10.0 cM window size. Five background control markers were chosen by forward-backward stepwise regression to reduce genetic background noise. A genome-wide LOD threshold was determined by 1,000 permutation tests at *p* < 0.05. The confidence interval for each QTL was defined using the 1.5-LOD support interval [52]. The phenotypic variation explained (PVE) by individual and combined QTLs were estimated via linear regression [5,34]. QTLs detected in multiple environments and by BLUP values were regarded as stable loci.

### 4.6. Epistatic Interaction Analysis

Epistatic interactions were tested using peak bin markers of identified QTLs. Only homozygous genotypes were retained, and heterozygous genotypes were treated as missing data to isolate additive-by-additive epistatic effects. Pairwise epistasis was examined using two-way ANOVA at *p* < 0.05. The PVE of each epistatic pair was estimated by comparing the full model (containing single-locus and two-locus interaction effects) with the reduced model (excluding interaction effects) [5,53,54].

### 4.7. Fine Mapping of the Major QTL on Chromosome 7

The major and stable QTL for ANT on chromosome 7 (*L4*) was fine-mapped using historical recombination events within the RIL population. Eight polymorphic SNPs within the initial QTL interval were used to genotype all lines. Haplotype analysis was performed to identify critical recombination breakpoints, and the target region was narrowed by comparing ANT contents between lines carrying contrasting haplotypes. Genes within the refined interval were annotated according to the B73 reference genome (RefGen_v4), and endosperm expression profiles were used to prioritize candidate genes [5,55].

### 4.8. Functional Validation of DXS2 Using MuDR Insertion Mutant

The *DXS2* MuDR transposon insertion mutant (Mu−*dxs2*) was acquired from the maize UniformMu Transposon Resource (https://www.maizegdb.org/uniformmu, accessed on 11 September 2016). The insertion site in the second exon was verified by PCR with two pairs of gene-specific primers (Table S1). Homozygous mutant and wild−type (WT) plants were grown under common field conditions, and ANT contents were measured in mature kernels.

### 4.9. RNA Sequencing

For transcriptomic profiling, endosperm tissues at 20 days after pollination (DAP) were collected from three biological replicates of WT and Mu-*dxs2* plants, with 20 endosperms per replicate. RNA-seq libraries were constructed and sequenced on an Illumina platform. Raw sequencing reads were subjected to quality control and filtering to remove low-quality reads and adapter contamination. The clean reads were aligned to the maize reference genome (V5), and the resulting read counts were used for differential expression analysis. Differentially expressed genes (DEGs) were identified using the DESeq2 package with thresholds of |log_2_FC| ≥ 1 and adjusted *p* value (false discovery rate, FDR) < 0.05 [56]. KEGG enrichment analysis was performed to characterize metabolic pathways affected by *DXS2* disruption [57,58].

## 5. Conclusions

In this study, we developed a high-resolution genetic map to detect the genetic architecture of maize kernel antheraxanthin (ANT) content, which is regulated by multiple QTLs and epistatic interactions across different environments. Fine mapping and mutant validation confirmed that the candidate gene *DXS2*, underlying the major-effect locus *L4*, directly regulates ANT content. Transcriptome analysis showed that loss of *DXS2* function leads to the downregulation of numerous genes in the MEP pathway and its downstream carotenoid synthesis and degradation pathways, thereby reducing ANT accumulation. These results provide important genetic resources and theoretical basis for improving the nutritional quality of maize.

## Figures and Tables

**Table 1:** Statistical analysis of ANT content in maize kernel among TM population.

Environment	Min	Max	Mean	Fold	Mean ± SD	H^2 a^
13Beijing	0.09	1.09	0.49	12.67	0.49 ± 0.17	
13Hainan	0.13	1.51	0.63	12.07	0.63 ± 0.27	
14Neimeng	0.03	0.95	0.40	27.77	0.40 ± 0.17	
BLUP	0.14	0.99	0.51	7.07	0.51 ± 0.16	0.86

^a^ H^2^ represents broad-sense heritability, which estimates the proportion of phenotypic variation attributable to total genetic effects.

**Table 2:** QTL results of ANT content in TM population.

Loci	QTL_ID	Repeats	Chr	Marker Interval	PK (cM)	G-Range (cM) ^a^	P-Range (Mb) ^b^	Non-Overlapped Region (Mb) ^c^	LOD	Add ^d^	PVE (%)
*L1*	*qANT1*	13BJ	1	SYN19155-PZE-101222331	122.40	114.30–124.40	259.78–273.45	259.8–273.4	5.66	0.10	9.11
*L2*	*qANT4*	14NM	4	PZE-104000008-PZE-104005531	0.00	0.00–4.60	0.03–1.46	0.03–1.46	4.23	−0.07	5.99
*L3*	*qANT5*	14NM	5	PZE-105090504-PZE-105113799	131.70	117.90–137.60	127.04–170.63	127.04–170.63	3.43	−0.06	4.81
*L4*	*qANT7-1*	13BJ	7	SYN3981-PZE-107018090	46.30	44.40–49.80	8.93–15.54	8.05–15.54	16.46	−0.14	34.45
	*qANT7-2*	13HN	7	SYN4526-SYN6901	46.30	42.90–49.80	8.05–10.70		12.93	−0.21	28.49
	*qANT7-3*	14NM	7	SYN3981-PZE-107018090	49.80	44.40–50.50	8.93–15.54		20.61	−0.14	37.46
	*qANT7-4*	BLUP	7	PZB02215.4-SYN6901	46.30	46.30–49.80	9.26–10.70		17.61	−0.13	30.69
*L5*	*qANT8-1*	13BJ	8	PZE-108109731-PZE-108118035	114.00	109.60–116.30	162.75–166.93	161.72–169.32	6.92	0.09	12.66
	*qANT8-2*	13HN	8	ZM012915-0492-PZE-108122589	118.00	111.60–122.20	164.03–169.32		3.81	0.12	7.10
	*qANT8-3*	14NM	8	SYN1030-PZE-108116305	111.60	109.00–114.00	162.22–166.11		5.37	0.07	8.13
	*qANT8-4*	BLUP	8	PZE-108108112-PUT-163a-76010623-3722	114.00	108.40–121.60	161.72–169.09		3.70	0.06	5.33

^a^ Genetic position range for the 1.5-LOD support interval. ^b^ Physical position range for the 1.5-LOD support interval in version 2. ^c^ Non-overlapped physical position for co-localization QTLs in version 2. ^d^ Positive values indicate that the teosinte allele has a positive effect on the corresponding trait. Negative values indicate that the maize allele has a positive effect on the corresponding trait.

## Data Availability

The original contributions presented in this study are included in the article and Supplementary Materials. The raw RNA-seq data have been deposited in the BIG Submission Portal (https://ngdc.cncb.ac.cn/gsub/submit/gsa/list, accessed on 8 July 2026) under accession number CRA046189.

## Supplementary Materials

The following supporting information can be downloaded at: https://www.mdpi.com/article/10.3390/ijms27156770/s1.
