# Supplementary material for: Genetic Dissection and Functional Validation of DXS2 Controlling Antheraxanthin Content in Maize Kernels
Source: Int J Mol Sci. 2026 Jul 28;27(15):6770. doi: 10.3390/ijms27156770 (PMC13467453; doi:10.3390/ijms27156770)
Supplement: Supplementary file 1 [file ijms-27-06770-s001.zip › ijms-4452892-supplementary.pdf]

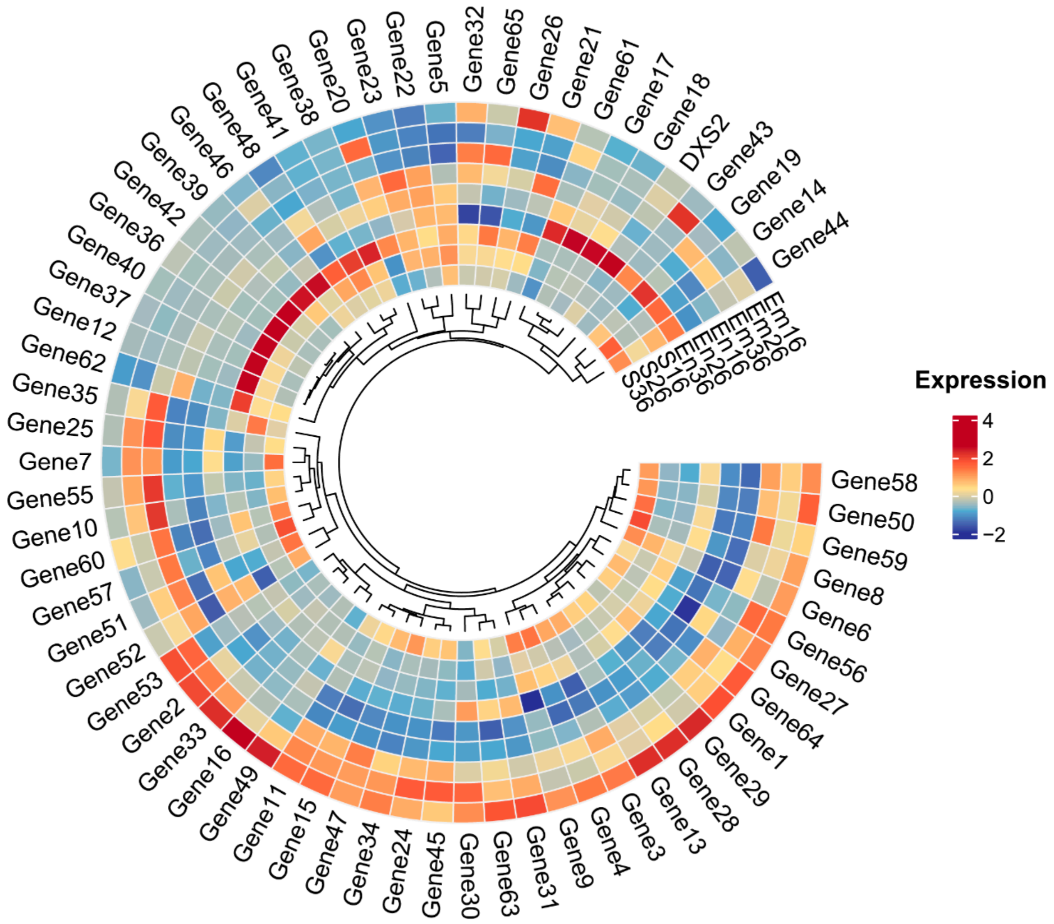


**Figure S1** Expression heatmap of 65 genes cover in the 6.3-Mb *L4* interval in embryo, endosperm and kernel.

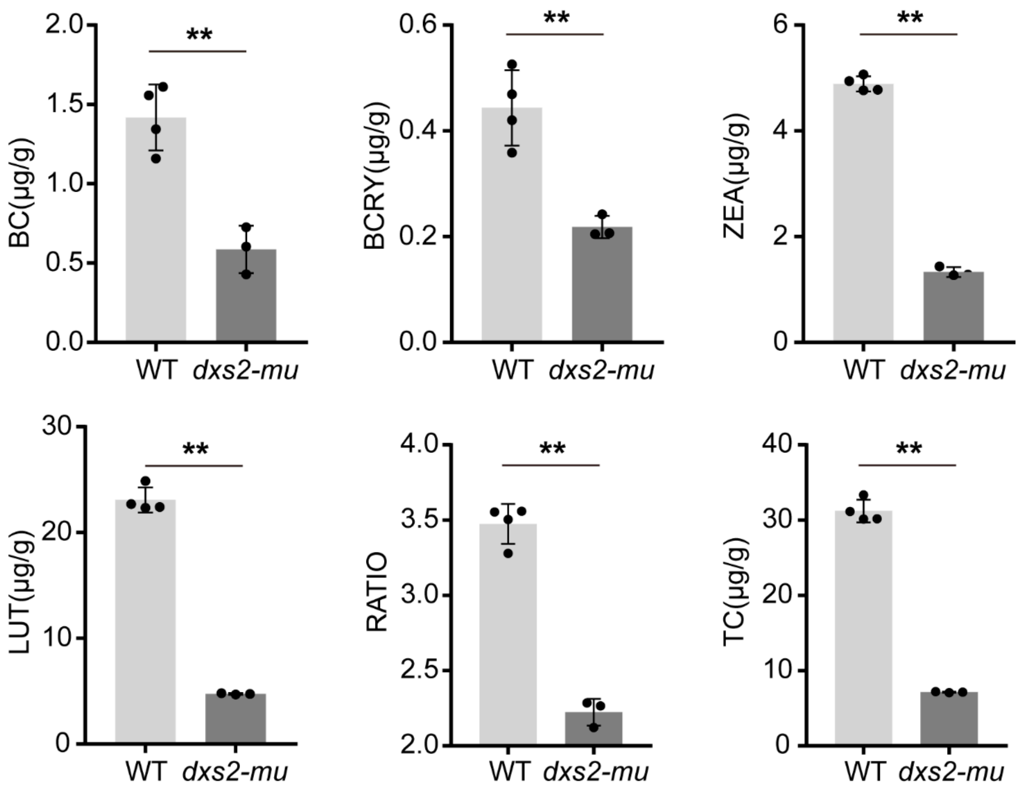


**Figure S2.** Phenotype of carotenoid-related traits in Wild-type and Mu-*dxs2* kernels.

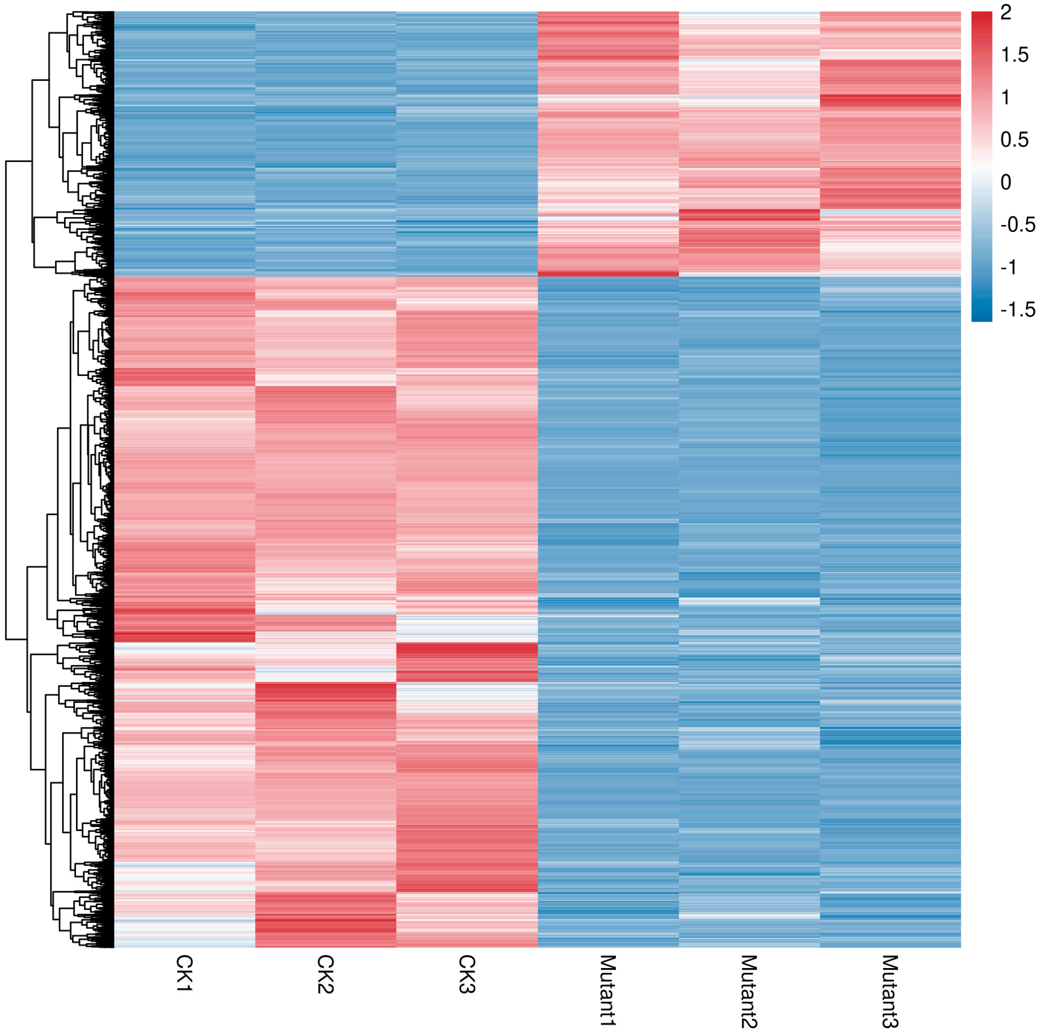


**Figure S3** Clustering heatmap of DEGs identified by RNA-seq.

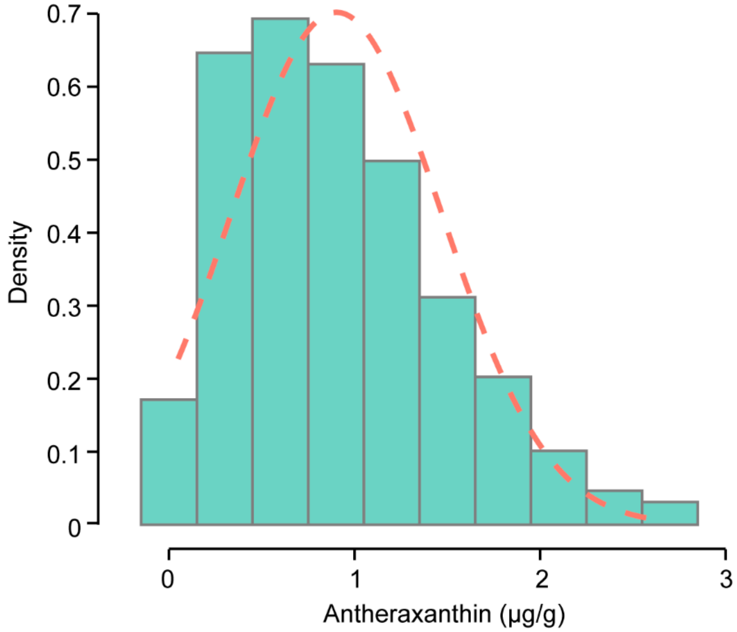


**Figure S4** Distribution of antheraxanthin (ANT) content in maize kernels in 456 maize inbred lines from the association mapping population.

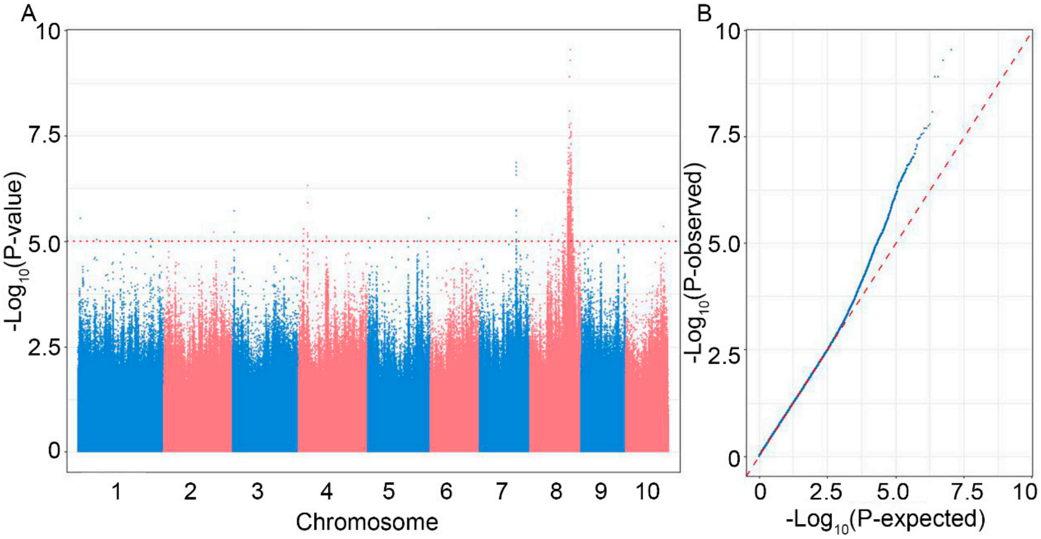


**Figure S5** GWAS results of ANT content in an association panel. (A) GWAS result and QQ plot (B) of ANT content in an association panel of 456 maize lines. The signal of *DXS2* was not detected.

Table S1 Primer sequences used to verify *DXS2* Mu insertion.

| Primer Name | Forward Sequence (5' to 3') |
|---|---|
| MuF | GGAAGATGATGGTTTCCAAG |
| MuR | ACACCTGCTTGTTGTCGTTC |
| TIR8 | CGCCTCCATTTCGTCGAATCCCCTS |

Table S2 Epistasis interaction between pairs of QTLs with additive effect.

| Environment | $QTL_i$ | $chr_i$ | Marker $interval_i$ | $P_i$[a] | $QTL_j$ | $chr_j$ | Marker $interval_j$ | $P_j$[a] | P-value[b] | PVE_add (%) | PVE_epi (%)[d] |
|---|---|---|---|---|---|---|---|---|---|---|---|
| 13BJ | *qANT1* | 1 | PZE-101217941 - PZE-101222233 | 122.4 | *qANT7-1* | 7 | PZB02215.4 - SYN6901 | 46 | 0.024 | 51.94 | 2.9 |
| 13HN | NA | NA | NA | NA | NA | NA | NA | NA | NA | 28.16 | NA |
| 14NM | *qANT5* | 5 | PZE-105099711 - PZE-105112279 | 131.7 | *qANT8-3* | 8 | PZE-108109731 - SYN22946 | 112 | 0.034 | 48.17 | 0.1 |
| BLUP | NA | NA | NA | NA | NA | NA | NA | NA | NA | 37.02 | NA |

[a] QTL genetic location on chromosome was estimated by WinQTLCart2.5.

[b] P-value estimated by a two-way ANOVA for the pairwise additive-by-additive epistatic interactions.

[c] Percentage of phenotypic variation explained by all additive effects of the mapped QTL in each environment.

[d] Percentage of phenotypic variation explained by individual epistatic effects of the mapped QTL.
